# Accurately Differentiating Between Patients With COVID-19, Patients With Other Viral Infections, and Healthy Individuals: Multimodal Late Fusion Learning Approach

**DOI:** 10.2196/25535

**Published:** 2021-01-06

**Authors:** Ming Xu, Liu Ouyang, Lei Han, Kai Sun, Tingting Yu, Qian Li, Hua Tian, Lida Safarnejad, Hengdong Zhang, Yue Gao, Forrest Sheng Bao, Yuanfang Chen, Patrick Robinson, Yaorong Ge, Baoli Zhu, Jie Liu, Shi Chen

**Affiliations:** 1 Department of Occupational Disease Prevention Jiangsu Provincial Center for Disease Control and Prevention Nanjing China; 2 Center for Global Health School of Public Health Nanjing Medical University Nanjing China; 3 Department of Public Health Sciences College of Health and Human Services University of North Carolina Charlotte Charlotte, NC United States; 4 Department of Orthopedics Union Hospital, Tongji Medical College Huazhong University of Science and Technology Wuhan China; 5 Department of Emergency Medicine The First Hospital of Nanjing Medical University Nanjing China; 6 Department of Medical Genetics School of Basic Medical Science Jiangsu Key Laboratory of Xenotransplantation Nanjing Medical University Nanjing China; 7 Department of Pediatrics Affiliated Kunshan Hospital of Jiangsu University Kunshan China; 8 Department of Acute Infectious Disease Control and Prevention Jiangsu Provincial Center for Disease Control and Prevention Nanjing China; 9 School of Medicine Stanford University Stanford, CA United States; 10 Department of Software and Information Systems College of Computing and Informatics University of North Carolina Charlotte Charlotte, NC United States; 11 Department of Computer Science Iowa State University Ames, IA United States; 12 Institute of HIV/AIDS/STI Prevention and Control Jiangsu Provincial Center for Disease Control and Prevention Nanjing China; 13 Department of Radiology Union Hospital, Tongji Medical College Huazhong University of Science and Technology Wuhan China; 14 School of Data Science University of North Carolina Charlotte Charlotte, NC United States

**Keywords:** COVID-19, machine learning, deep learning, multimodal, feature fusion, biomedical imaging, diagnosis support, diagnosis, imaging, differentiation, testing, diagnostic

## Abstract

**Background:**

Effectively identifying patients with COVID-19 using nonpolymerase chain reaction biomedical data is critical for achieving optimal clinical outcomes. Currently, there is a lack of comprehensive understanding in various biomedical features and appropriate analytical approaches for enabling the early detection and effective diagnosis of patients with COVID-19.

**Objective:**

We aimed to combine low-dimensional clinical and lab testing data, as well as high-dimensional computed tomography (CT) imaging data, to accurately differentiate between healthy individuals, patients with COVID-19, and patients with non-COVID viral pneumonia, especially at the early stage of infection.

**Methods:**

In this study, we recruited 214 patients with nonsevere COVID-19, 148 patients with severe COVID-19, 198 noninfected healthy participants, and 129 patients with non-COVID viral pneumonia. The participants’ clinical information (ie, 23 features), lab testing results (ie, 10 features), and CT scans upon admission were acquired and used as 3 input feature modalities. To enable the late fusion of multimodal features, we constructed a deep learning model to extract a 10-feature high-level representation of CT scans. We then developed 3 machine learning models (ie, k-nearest neighbor, random forest, and support vector machine models) based on the combined 43 features from all 3 modalities to differentiate between the following 4 classes: nonsevere, severe, healthy, and viral pneumonia.

**Results:**

Multimodal features provided substantial performance gain from the use of any single feature modality. All 3 machine learning models had high overall prediction accuracy (95.4%-97.7%) and high class-specific prediction accuracy (90.6%-99.9%).

**Conclusions:**

Compared to the existing binary classification benchmarks that are often focused on single-feature modality, this study’s hybrid deep learning-machine learning framework provided a novel and effective breakthrough for clinical applications. Our findings, which come from a relatively large sample size, and analytical workflow will supplement and assist with clinical decision support for current COVID-19 diagnostic methods and other clinical applications with high-dimensional multimodal biomedical features.

## Introduction

COVID-19 is an emerging major biomedical challenge for the entire health care system [1]. Compared to severe acute respiratory syndrome (SARS) and Middle East respiratory syndrome (MERS), COVID-19 has much higher infectivity. COVID-19 has also spread much faster across the globe than other coronavirus diseases. Although COVID-19 has a relatively lower case fatality rate than SARS and MERS, the overwhelmingly large number of diagnosed COVID-19 cases, as well as the many more undiagnosed COVID-19 cases, has endangered health care systems and vulnerable populations during the COVID-19 pandemic. Therefore, the early and accurate detection and intervention of COVID-19 are key in effectively treating patients, protecting vulnerable populations, and containing the pandemic at large.

Currently, the gold standard for the confirmatory diagnosis of COVID-19 is based on molecular quantitative real-time polymerase chain reaction (qRT-PCR) and antigen testing for the disease-causing SARS-CoV-2 virus [2-4]. Although these tests are the gold standard for COVID-19 diagnosis, they suffer from various practical issues, including reliability, resource adequacy, reporting lag, and testing capacity across time and space [5]. To help frontline clinicians diagnose COVID-19 more effectively and efficiently, other diagnostic methods have also been explored and used, including medical imaging (eg, X-ray scans and computed tomography [CT] scans [6]), lab testing (eg, various blood biochemistry analyses [7-10]), and identifying common clinical symptoms [11]. However, these methods do not directly detect the disease-causing SARS-CoV-2 virus or the SARS-CoV-2 antigen. Therefore, these methods do not have the same conclusive power that confirmatory molecular diagnostic methods have. Nevertheless, these alternative methods help clinicians with inadequate resources detect COVID-19, differentiate patients with COVID-19 from patients without COVID-19 and noninfected individuals, and triage patients to optimize health care system resources [12,13]. When applied appropriately, these supplementary methods, which are based on alternative biomedical evidence, can help mitigate the COVID-19 pandemic by accurately identifying patients with COVID-19 as early as possible.

Currently, CT scans can be analyzed to differentiate patients with COVID-19, especially those in a severe clinical state, from healthy people or patients with non-COVID infections. Patients with COVID-19 usually present the typical ground-glass opacity (GGO) characteristic on CT images of the thoracic region. A recent study has reported a 98% COVID-19 detection rate based on a 51-patient sample without a comparison group [14]. Detection rates that ranged between 60% and 93% were also reported in another study on 1014 participants with a comparison group [15]. Furthermore, the recent advances in data-driven deep learning (DL) methods, such as convolutional neural networks (CNNs), have demonstrated the ability to detect COVID-19 in patients. On February 2020, Hubei, China adopted CT scans as the official clinical COVID-19 diagnostic method in addition to molecular confirmatory diagnostic methods for COVID-19, in accordance with the nation’s diagnosis and treatment guidance [2]. However, the effectiveness of using DL methods to further differentiate SARS-CoV-2 infection from clinically similar non-COVID viral infections still needs to be explored and evaluated.

With regard to places where molecular confirmatory diagnoses are not immediately available, symptoms are often used for quickly evaluating presumed patients’ conditions and supporting triage [13,16,17]. Checklists have been developed for self-evaluating the risk of developing COVID-19. These checklists are based on clinical information, including symptoms, preexisting comorbidities, and various demographic, behavioral, and epidemiological factors. However, these clinical data are generally used for qualitative purposes (eg, initial assessment) by both the public and clinicians [18]. Their effectiveness in providing accurate diagnostic decision support is largely underexplored and unknown.

In addition to biomedical imaging and clinical information, recent studies on COVID-19 have shown that laboratory testing, such as various blood biochemistry analyses, is also a feasible method for detecting COVID-19 in patients, with reasonably high accuracy [19,20]. The rationale is that the human body is a unity. When people are infected with SARS-CoV-2, the clinical consequences can be observed not only from apparent symptoms, but also from hematological biochemistry changes. Due to the challenge of asymptomatic SARS-CoV-2 infection, other types of biomedical information, such as lab testing results, can be used to provide alternative and complementary diagnostic decision support evidence. It is possible that our current definition and understanding of asymptomatic infection can be extended with more intrinsic, quantitative, and subtle medical features, such as blood biochemistry characteristics [21,22].

Despite the tremendous advances in obtaining alternative and complementary diagnostic evidence for COVID-19 (eg, CT scans, chest X-rays, clinical information, and various blood biochemistry characteristics), there are still substantial clinical knowledge gaps and technical challenges that hinder our efforts in harnessing the power of various biomedical data. First, most recent studies have usually focused on one of the multiple modalities of diagnostic data, and these studies have not considered the potential interactions between and added interpretability of these modalities. For example, can we use both CT scan and clinical information to develop a more accurate COVID-19 decision support system [23]? As stated earlier, the human body acts as a unity against SARS-CoV-2 infection. Biomedical imaging and clinical approaches can be used to evaluate different aspects of the clinical consequences of COVID-19. By combining the different modalities of biomedical information, a more comprehensive characterization of COVID-19 can be achieved. This is referred to as multimodal biomedical information research.

Second, while there are ample accurate DL algorithms/models/ tools, especially in biomedical imaging, most of them focus on differentiating patients with COVID-19 from noninfected healthy individuals. A moderately trained radiologist can differentiate CT scans of patients with COVID-19 from those of healthy individuals with high accuracy, making current efforts in developing supplicated DL algorithms not clinically useful for solving the binary classification problem [14]. The more critical and urgent clinical issue is not only being able to differentiate patients with COVID-19 from noninfected healthy individuals, but also being able to differentiate SARS-CoV-2 infection from non-COVID viral infections [24,25]. Patients with non-COVID viral infection present with GGO in their CT scans of the thoracic region as well. Therefore, the specificity of GGO as a diagnostic criterion of COVID-19 is low [15]. In addition, patients with nonsevere COVID-19 and patients with non-COVID viral infection share several common symptoms, which are easy to confuse [26]. Therefore, for frontline clinicians, effectively differentiating nonsevere COVID-19 from non-COVID viral infection is a challenging task without readily available and reliable confirmatory molecular tests at admission. Incorrectly diagnosing severe COVID-19 as nonsevere COVID-19 may result in missing the critical window of intervention. Similarly, differentiating asymptomatic and presymptomatic patients, including those with nonsevere COVID-19, from noninfected healthy individuals is another major clinical challenge [27]. Incorrectly diagnosing patients without COVID-19 or healthy individuals and treating them alongside patients with COVID-19 will substantially increase their risk of exposure to the virus and result in health care–associated infections. There is an urgent need for a multinomial classification system that can detect patients with COVID-19, including patients with asymptomatic COVID-19, patients with non-COVID viral infection, and healthy individuals, all at once, rather than a system that analyzes several independent binary classifiers in parallel [28].

The third major challenge addresses the computational aspect of harnessing the power of various biomedical data. Due to the novelty of the COVID-19 pandemic, human clinicians have varying degrees of understanding and experience with regard to COVID-19, which can lead to inconsistencies in clinical decision making. Harnessing the power of multimodal biomedical information from combined imaging, clinical, and lab testing data can be the basis of a more objective, data-driven, analytical framework. In theory, such a framework can provide a more comprehensive understanding of COVID-19 and a more accurate decision support system that can differentiate between patients with severe or nonsevere COVID-19, patients with non-COVID viral infection, and healthy individuals all at once. However, biomedical imaging data, such as CT data, with a high-dimensional feature space do not integrate well with low-dimensional clinical and lab testing data. Current studies have usually only described the association between biomedical imaging and clinical features [15,29-33], and the potential power of an accurate decision support tool has not been reported. Technically, CT scans are usually processed with DL methods, including the CNN method, independently from other types of biomedical data processing methods. Low-dimensional clinical and lab testing data are usually analyzed with traditional hypothesis-driven methods (eg, binary logistic regression or multinomial classification) or other non-DL machine learning (ML) methods, such as the random forest (RF), support vector machine (SVM), and k-nearest neighbor (kNN) methods. The huge discrepancy of feature space dimensionality between CT scan and clinical/lab testing data makes multimodal fusion (ie, the direct combination of the different aspects of biomedical information) especially challenging [34].

To fill these knowledge gaps and overcome the technical challenge of effectively analyzing multimodal biomedical information, we propose the following study objective: we aimed to clinically and accurately differentiate between patients with nonsevere COVID-19, patients with severe COVID-19, patients with non-COVID viral pneumonia, and healthy individuals all at once. To successfully fulfill this much-demanded clinical objective, we developed a novel hybrid DL-ML framework that harnesses the power of a wide array of complex multimodal data via feature late fusion. The clinical objective and technical approach of this study synergistically complements each other to form the basis of an accurate COVID-19 diagnostic decision support system.

## Methods

### Participant Recruitment

We recruited a total of 362 patients with confirmed COVID-19 from Wuhan Union Hospital between January 2020 and March 2020 in Wuhan, Hubei Province, China. COVID-19 was confirmed based on 2 independent qRT-PCR tests. For this study, we did not aggregate patients with COVID-19 under the same class because the clinical characteristics of nonsevere and severe COVID-19 were distinct. Patients’ COVID-19 status was confirmed upon admission. The recruited patients were further categorized as being in severe (n=148) or nonsevere (n=214) clinical states based on their prognosis at 7-14 days after initial admission. This step ensured the development of an early detection system for when the initial conditions of patients with COVID-19 were not severe upon admission. Patients in the severe state group were identified by having 1 of the following 3 clinical features: (1) respiratory rate>30 breaths per minute, (2) oxygen saturation<93% at rest, and (3) arterial oxygen partial pressure/fraction of inspired oxygen<300 mmHg (ie, 40 kPa). These clinical features are based on the official COVID-19 Diagnosis and Treatment Plan from the National Health Commission of China [2], as well as guidelines from the American Thoracic Society [35]. The noninfected group included 198 healthy individuals without any infections. These participants were from the 2019 Hubei Provincial Centers for Disease Control and Prevention regular annual physical examination cohort. This group represented a baseline healthy group, and they were mainly used as a comparison group for patients with nonsevere COVID-19, especially those who presented with inconspicuous clinical symptoms.

In order to differentiate patients with COVID-19, especially those with nonsevere COVID-19, from patients with clinically similar non-COVID viral infection, we also included another group of 129 patients diagnosed with non-COVID viral pneumonia in this study. It should be noted that the term “viral pneumonia” was an umbrella term that included diseases caused by more than 1 type of virus, such as the influenza virus and adenovirus. However, in clinical practice, it would be adequate to detect and differentiate between SARS-CoV-2 infection and non-COVID viral infections for initial triaging. Therefore, we recruited 129 participants with confirmed non-COVID viral infection from Kunshan Hospital, Suzhou, China. The reality was that most health care resources were optimized for COVID-19, and some patients who presented with COVID-19–like symptoms or GGOs were clinically diagnosed with COVID-19 without the use of confirmatory qRT-PCR tests in Hubei, especially during February 2020. Therefore, it was not possible to recruit participants with non-COVID viral infection in Hubei during the same period that we recruited patients with COVID-19.

In summary, the entire study sample was comprised of the following 4 mutually exclusive multinomial participant classes: severe COVID-19 (n=148), nonsevere COVID-19 (n=214), non-COVID viral infection (n=129), and noninfected healthy (n=198). This study was conducted in full compliance with the Declaration of Helsinki. This study was rigorously evaluated and approved by the institutional review board committees of Jiangsu Provincial Center for Disease Control and Prevention (approval number JSJK2020-8003-01). All participants were comprehensively told about the details of the study. All participants signed a written informed consent form before being admitted.

### Medical Feature Selection and Description

**Figure 1:**
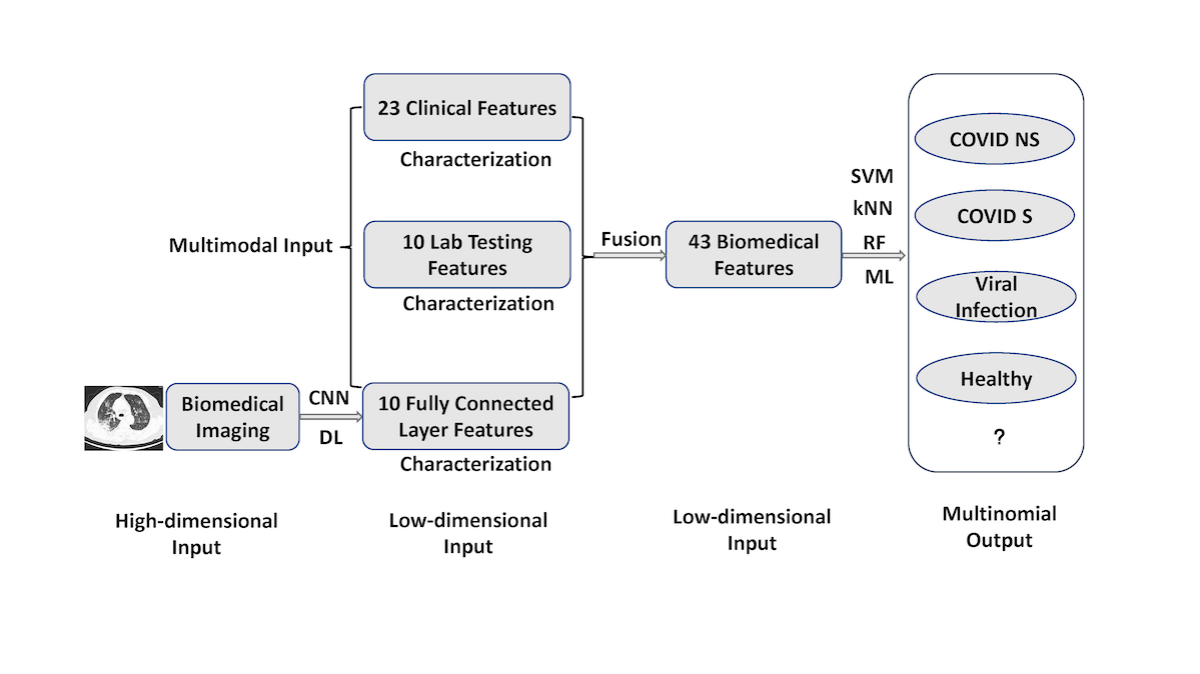
Multimodal feature late fusion and multinomial classification workflow. A deep learning convolutional neural network was applied to computed tomography images for representation learning and extracting 10 features from a customized fully connected layer. These 10 features were merged with other modality data through feature late fusion. In the machine learning stage of the workflow, each of the 3 machine learning models (ie, the support vector machine, k-nearest neighbor, and random forest models) worked independently to provide their respective outputs. kNN: k-nearest neighbor; ML: machine learning; RF: random forest; SVM; support vector machine.

Patient participants, including those in the severe COVID-19, nonsevere COVID-19, and non-COVID viral infection classes, were screened upon initial admission into hospitals. Their clinical information, including preexisting comorbidities, symptoms, demographic characteristics, epidemiological characteristics, and other clinical data, were recorded. For the noninfected healthy class, participants’ clinical data were extracted from the Hubei Provincial Centers for Disease Control and Prevention physical examination record system. Patient-level sensitive information, including name and exact residency, were completely deidentified. After comparing the different classes, the following 23 clinical features were selected for this study: smoking history, hypertension, type-2 diabetes, cardiovascular disease (ie, any type), chronic obstructive pulmonary disease, fever, low fever, medium fever, high fever, sore throat, coughing, phlegm production, headache, feeling chill, muscle ache, feelings of fatigue, chest congestion, diarrhea, loss of appetite, vomiting, old age (ie, >50 years; dichotomized and encoded as old), and gender. These clinical data were dichotomized as either having the condition (score=1) or not having the condition (score=0) (Figure 1). It should be noted that several clinical features, especially symptoms, were self-reported by the patients. A more comprehensive definition and description of clinical features are provided in Multimedia Appendix 1. The prevalence (ie, the number of participants that have a given feature over the total number of participants in the class) of each clinical feature was computed across the 4 classes. For the 0-1 binary clinical features, a pairwise z-test was applied to detect any substantial differences in the prevalence (ie, proportion) of these features between classes.

The lab testing features were extracted from participants’ electronic health records (Figure 1). Only the features from lab tests that were performed at the time of admission were included. Noninfected healthy participants’ blood samples were taken during their annual physical examination. We selected lab testing features that were present in at least 90% of participants in any of the 4 classes (ie, severe COVID-19, nonsevere COVID-19, non-COVID viral infection, and noninfected healthy). After screening, the following 10 features were included: white blood cell count, hemoglobin level, platelet count, neutrophil count, neutrophil percent, lymphocyte count, lymphocyte percent, C-reactive protein level, total bilirubin level, and creatine level. Features in the lab testing modality all had continuous numeric values, which were different from the 0-1 binary values in the clinical feature modality. The distributions of these lab testing features were compared across the 4 classes by using a 2-sided Kolmogorov-Smirnov test. In addition, we also applied the Kruskal-Wallis test for multiple comparisons across the 4 classes for the top 3 most differentiating features, which were identified later by an ML workflow. The Kolmogorov-Smirnov test was applied during initial screening to investigate whether the values of the same biomedical feature were distributed differently between 2 classes. The nonparametric Kruskal-Wallis test was chosen because it could rigorously compare classes and provide robust results for nonnormal data. The test was able to accommodate more than 2 classes (ie, multinomial classes) in this study.

Each participant underwent CT scans of the thoracic region in the radiology department. Toshiba Activion 16 multislice CT scanners were used to perform CT scanning at around 120 kVp with a tube current of 50 mA. We obtained 50 CT images per scan, and each image had the following characteristics: slice thickness=2 mm, voxel size=1.5 mm, and image resolution=512×512 pixels. Each participant underwent an average of 50 CT scans. The total number of CT images obtained in this study was over 30,000. CT images were archived and presented as DICOM (Digital Imaging and Communications in Medicine) images for DL.

### The Multinomial Classification Objective

The main research goal of this study was to accurately differentiate between patients with severe COVID-19, patients with nonsevere COVID-19, patients with non-COVID viral infection, and noninfected healthy individuals from a total of N participants all at once. Therefore, a formula was developed to address the multinomial output classification problem. The following equation uses 1 of the 4 mutually exclusive output classes (ie, H=noninfected healthy, V=non-COVID viral pneumonia, NS=nonsevere COVID-19, and S=severe COVID-19) of an individual (ie, i), as follows:


f(X_c_,X_l_,X_m_)*_i_* = {H,V,NS,S}, i = 1...N **(1)**

In this equation, the inputs were individuals’ (ie, i) multimodal features of binary clinical information (ie, X_c_), continuous lab test results (ie, X_l_), and CT imaging (ie, X_m_). The major advantage of our study was that we were able to classify 4 classes all at once, instead of developing several binary classifiers in parallel.

### The Hybrid DL-ML Approach: Feature Late Fusion

As stated earlier, the voxel level of CT imaging data does not integrate well with low-dimensional clinical and lab testing features. In this study, we proposed a feature late fusion approach via the use of hybrid DL and ML models. Technically, DL is a type of ML that uses deep neural networks (eg, CNNs are a type of deep neural network). In this study, we colloquially used the term “machine learning” to refer to more traditional, non-DL types of ML (eg, RF ML), in contrast with DL that focuses on deep neural networks. An important consideration in the successful late fusion of multimodality features is the representation learning of the high-dimensional CT features.

For each CT scan of each participant, we constructed a customized residual neural network (ResNet) [36-39], which is a specific architecture for DL CNNs. A ResNet is considered a mature CNN architecture with relatively high performance across different tasks. Although other CNN architectures exist (eg, EfficientNet, VGG-16, etc), the focus of this study was not to compare different architectures. Instead, we wanted to deliver the best performance possible with a commonly used CNN architecture (ie, ResNet) for image analysis.

By constructing a ResNet, we were able to transform the voxel-level imaging data into a high-level representation with significantly fewer features. After several convolution and max pooling layers, the ResNet reached a fully connected (FC; ie, FC1 layer) layer before the final output layer, thereby enabling the delivery of actual classifications. In the commonly used ResNet architecture, the FC layer is a 1×512 vector, which is relatively closer in dimensionality to clinical information (ie, 1×23 vector) and lab testing (ie, 1×10 vector) feature modalities. However, the original FC layer from the ResNet was still much larger than the other 2 modalities. Therefore, we added another FC layer (ie, FC2 layer) after the FC1 layer, but before the final output layer. In this study, the FC2 layer was set to have a 1×10 vector dimension (ie, 10 elements in the vector) to match the dimensionality of the other 2 feature modalities. Computationally, the FC2 layer served as the low-dimensional, high-level representation of the original CT scan data. The distributions of the 10 features extracted from the ResNet in the FC2 layer were compared across the 4 classes with the Kolmogorov-Smirnov test. The technical details of this customized ResNet architecture are provided in Multimedia Appendix 2.

Once low-dimensional high-level features were extracted from CT data via the ResNet CNN, we performed multimodal feature fusion. The clinical information, lab testing, and FC2 layer features of each participant (ie, i) were combined into a single 1× 43 (ie, 1×[23+10+10]) row vector. The true values of the output were the true observed classes of the participants. Technically, the model would try to predict the outcome as accurately as it could, based on the observed classes.

### The Hybrid DL-ML Approach: Modeling

After deriving the feature matrix, we applied ML models for the multinomial classification task. In this study, 3 different types of commonly used ML models were considered, as follows: the RF, SVM, and kNN models. An RF model is a decision-tree–based ML model, and the number of tree hyperparameters was set at 10, which is a relatively small number compared to the number of input features needed to avoid potential model overfitting. Other RF hyperparameters in this study included the Gini impurity score to determine tree split, at least 2 samples to split an internal tree, and at least 1 sample at a leaf node. All default hyperparameter settings, including those of the SVM and RF models, were based on the scikit-learn library in Python. An SVM model is a model of maximum hyperplane and L-2 penalty; radial basis function kernels and a gamma value of 1/43 (ie, the inverse of the total number of features) were used as hyperparameter values in this study. kNN is a nonparametric instance-based model; the following hyperparameter values were used in this study: k=5, uniform weights, tree leaf size=30, and p=2. These 3 models are technically distinct types of ML models. We aimed to investigate whether specific types of ML models and multimodal feature fusion would contribute to developing an accurate COVID-19 classifier for clinical decision support.

We evaluated each respective ML model with 100 independent runs. Each run used a different randomly selected dataset comprised of 80% of the original data for training, and the remaining 20% of data were used to test and validate the model. Performing multiple runs instead of a single run revealed how robust the model was, despite system stochasticity. The 80%-20% split of the original data for separate training and testing sets also ensured that potential model overfitting and increased model generalizability could be avoided. In addition, RF models use bagging for internal validation based on out-of-bag errors (ie, how the “tree” would split out in the “forest” model).

After each run, important ML performance metrics, including accuracy, sensitivity, precision, and F1 score, were computed for the test set. We reported the overall performance of the ML models first. These different metrics evaluated ML models based on different aspects. In this study, we also considered 3 different approaches for calculating the overall performance of multinomial outputs, as follows: a micro approach (ie, the one-vs-all approach), a macro approach (ie, unweighted averages; each of the 4 classes were given the same 25% weights), and a weighted average approach based on the percentage of each class in the entire sample.

In addition, because the output in this study was multinomial instead of binary, each class had its own performance metrics. We aggregated these performance metrics across 100 independent runs, determined each metric’s distribution, and evaluated model robustness based on these distributions. If ML performance metrics in the testing set had a small variation (ie, small standard errors), then the model was considered robust against model input changes, thereby allowing it to reveal the intrinsic pattern of the data. This was because in each run, a different randomly selected dataset (ie, 80% of the original data) was selected to train the model.

An advantage that the RF model had over SVM and kNN models was that it had relatively clearer interpretability, especially when interpreting feature importance. After developing the RF model based on the training set, we were able to rank the importance of input features based on their corresponding Gini impurity score from the RF model [40,41]. It should be noted that only the training set was used to compute Gini impurity, not the test set. We then assessed the top contributing features’ clinical relevance to COVID-19.

We also developed and evaluated the performance of single-modality (ie, using clinical information, lab testing, and CT features individually) ML models. The performance results were used as baseline conditions. The models’ performance results were then compared to the multimodal classifications to demonstrate the potential performance gain of the feature fusion of different feature modalities. In this study, each individual ML model (ie, the RF, SVM, and kNN models) was independently evaluated, and the respective results were reported, without combining the prediction of the final output class.

The deep learning CNN and late fusion machine learning codes were developed in Python with various supporting packages, such as scikit-learn.

## Results

### Clinical Characterization of the 4 Classes

Detailed demographic, clinical, and lab testing results among four classes were provided in supplementary Table S1. We compared clinical features across the 4 classes. The prevalence of each feature in all 4 classes is shown in Multimedia Appendix 3. In general, most clinical features varied substantially between the nonsevere COVID-19, severe COVID-19, and non-COVID viral pneumonia classes. It should be noted that all symptom feature values, except gender and age group (ie, >50 years) values, in the noninfected healthy class were set to 0, so that they could be used as a reference. Based on the 2-sample z-test of proportions, the nonsevere COVID-19 and severe COVID-19 classes differed significantly (*P*<.05) in 10 out of 22 symptom features, including comorbidities such as hypertension (*P*<.001), diabetes (*P*<.001), cardiovascular diseases (*P*<.001), and chronic obstructive pulmonary disease (*P*=.005). The nonsevere COVID-19 and non-COVID viral infection classes differed significantly in 12 features, including smoking habit (*P*<.001), fever (*P*<.001), and sore throat (*P*=.002). However, the nonsevere COVID-19 and non-COVID viral infection classes did not differ significantly in terms of comorbidities. The severe COVID-19 and non-COVID viral infection classes differed significantly in 16 out of 22 features, making these 2 classes the most distinct in terms of symptoms. These results showed that the prevalence of clinical features differed substantially between the classes. The complete z-test results for each clinical feature in each pair of classes are provided in Multimedia Appendix 1.

**Figure 2:**
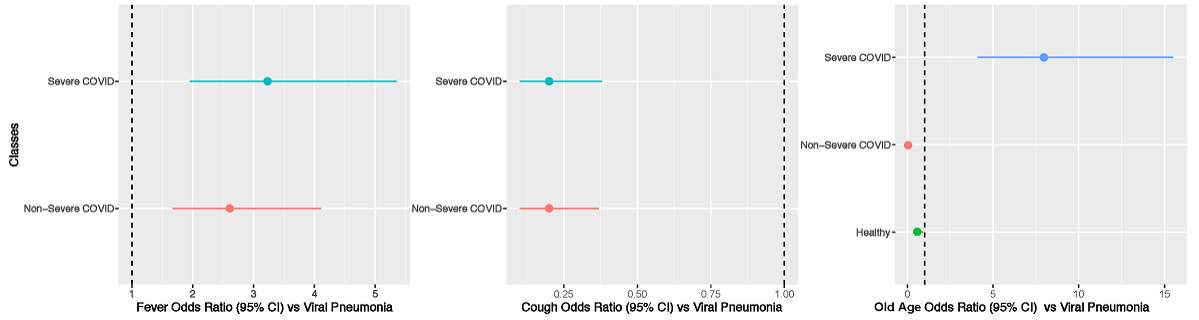
Forest plot of the top 3 differentiating clinical features. Viral pneumonia was used as the reference class during comparisons and the calculation of odds ratios. The noninfected healthy class had no individuals with fevers or coughs. Therefore, these individuals were not included in the first 2 graphs (ie, the left and middle graphs). The error bars represent variation in estimated odds ratios, not the original feature variations.

In addition, based on the ML RF analysis, the top 3 differentiating clinical features were fever, coughing, and old age (ie, >50 years). For fever and coughing, we used the non-COVID viral infection class as the reference and constructed 2×2 contingency tables for the nonsevere COVID-19 and non-COVID viral infection classes, and the severe COVID-19 and non-COVID viral infection classes. The odds ratios and 95% confidence intervals for the forest plot are shown in Figure 2. Compared to patients in the non-COVID viral infection class, patients in both the nonsevere and severe COVID-19 classes were more likely to develop fever (ie, >37°C). In addition, based on the forest plot, patients with severe COVID-19 also experienced more fevers than patients with nonsevere COVID-19. Therefore, fever was one of the major determining factors of differentiating between multiple classes. Furthermore, patients with nonsevere COVID-19 (*P*<.001) and patients with severe COVID-19 (*P*<.001) reported significantly less coughing than the patients with non-COVID viral infection (Figure 2). There were no statistically significant differences between the nonsevere and severe COVID-19 classes in terms of clinical features. With regard to the old age feature, we included the severe COVID-19, nonsevere COVID-19, and noninfected healthy classes in the analysis because the prevalence of old age in the noninfected healthy class was not 0. The forest plot for this analysis is shown in Figure 2. Patients with severe COVID-19 were significantly older than patients with non-COVID viral infection, while patients with nonsevere COVID-19 and noninfected healthy individuals were younger than patients with non-COVID viral infection. These differences in clinical features between the 4 classes could pave the way toward a data-driven ML model.

### Differences in Lab Testing Features Between the 4 Classes

With regard to the continuous lab testing features, we calculated and compared the exact distributions among the 4 classes. The boxplots for each lab testing feature across the 4 classes are provided in Multimedia Appendix 4. In general, the 4 classes differed substantially across many lab testing features. Based on the 2-sided Kolmogorov-Smirnov test results, the nonsevere and severe COVID-19 classes were only similar in hemoglobin level (HGB *P*=.74) and platelet count (PLT *P*=.61). These 2 classes differed significantly in the remaining 8 lab testing features (WBC *P*=.02; NE% *P*<.001; NE *P*<.001; LY% *P*<.001; LY *P*=.002; CRP *P*<.001; TBIL *P*=.001; CREA *P*<.001;) . In other words, the lab testing features of patients with severe or nonsevere COVID-19 had distinct distributions. Similarly, the nonsevere COVID-19 and noninfected healthy classes were only similar in creatine level; the nonsevere COVID-19 and non-COVID viral infection classes were only similar in hemoglobin level (*P*=.65), platelet count (*P*=.14), and total bilirubin level (*P*=.09); the severe COVID-19 and noninfected healthy classes were only similar in total bilirubin level (*P*=.24); the severe COVID-19 and non-COVID viral infection classes were only similar in hemoglobin level (*P*=.11) and neutrophil count (*P*=.08); and the non-COVID viral infection and noninfected healthy classes were only similar in white blood cell count (*P*=.70). The complete Kolmogorov-Smirnov test results for each lab testing feature in each pair of classes are provided in Multimedia Appendix 1.

**Figure 3:**
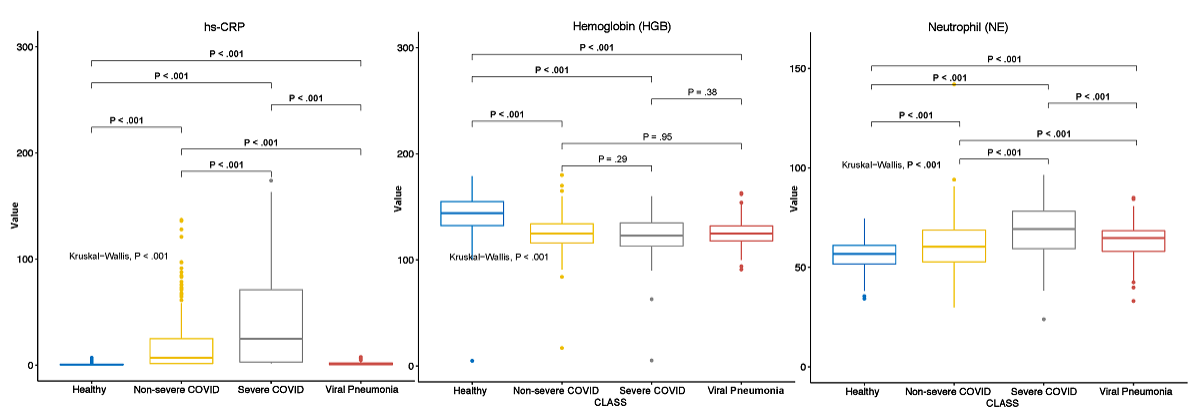
Multiple comparisons of the top differentiating lab testing features. hs-CRP: high-sensitivity C-reactive protein.

Based on the RF model, the 3 most influential differentiating features were C-reactive protein level, hemoglobin level, and neutrophil count. The distribution of C-reactive protein level among the 4 classes are provided in the boxplot in Figure 3. In addition to the Kolmogorov-Smirnov test, which did not account for multiple comparisons between classes, further pairwise comparisons were performed with the nonparametric Kruskal-Wallis H test. Each of the 6 pairs used in the Kruskal-Wallis H test, as well as the overall Kruskal-Wallis test, showed significant differences between each class. The distribution of hemoglobin levels is shown in Figure 3. Although the noninfected healthy class differed significantly from the nonsevere COVID-19, severe COVID-19, and non-COVID viral infection class in terms of hemoglobin level, the other 3 pairs did not show statistically significant differences in lab testing features. The distribution of neutrophil count is shown in Figure 3. All pairwise comparisons and the overall Kruskal-Wallis test showed significant differences between classes in terms of lab testing features.

### CT Differences Between the 4 Classes Based on High-Level CNN Features

**Figure 4:**
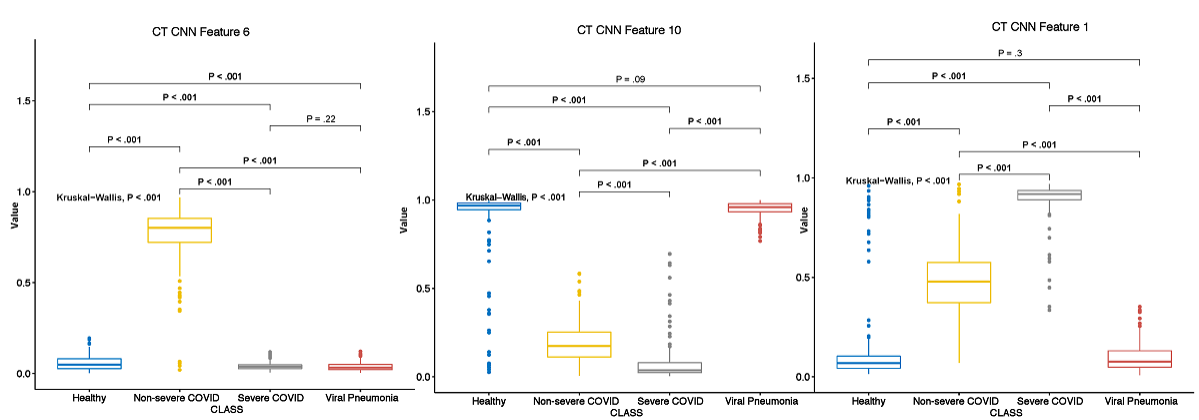
Multiple comparisons of the top differentiating CT features in the CNN. CNN: convolutional neural network; CT: computed tomography.

We analyzed the FC2 layer features from the ResNet CNN in relation to the 4 classes. The corresponding boxplot is shown in Multimedia Appendix 5. The 2-sided Kolmogorov-Smirnov tests showed significant differences between every pair of classes in almost all 10 CT features in the FC2 layer. The only exceptions were feature 6 (ie, CNN6) between the severe COVID-19 and non-COVID viral infection classes and features 1, 4, and 5 between the noninfected healthy and non-COVID viral infection classes (Multimedia Appendix 6). Based on the RF model results, features 1, 6, and 10 were the 3 most critical features in the FC2 layer with regard to multinomial classification. Further Kruskal-Wallis tests were performed for these 3 features, and the results are shown in Figure 4. These results showed that developing an accurate classifier based on the CNN representation of high-level features is possible.

### Accurate Multimodal Model for COVID-19 Multinomial Classification

**Figure 5:**
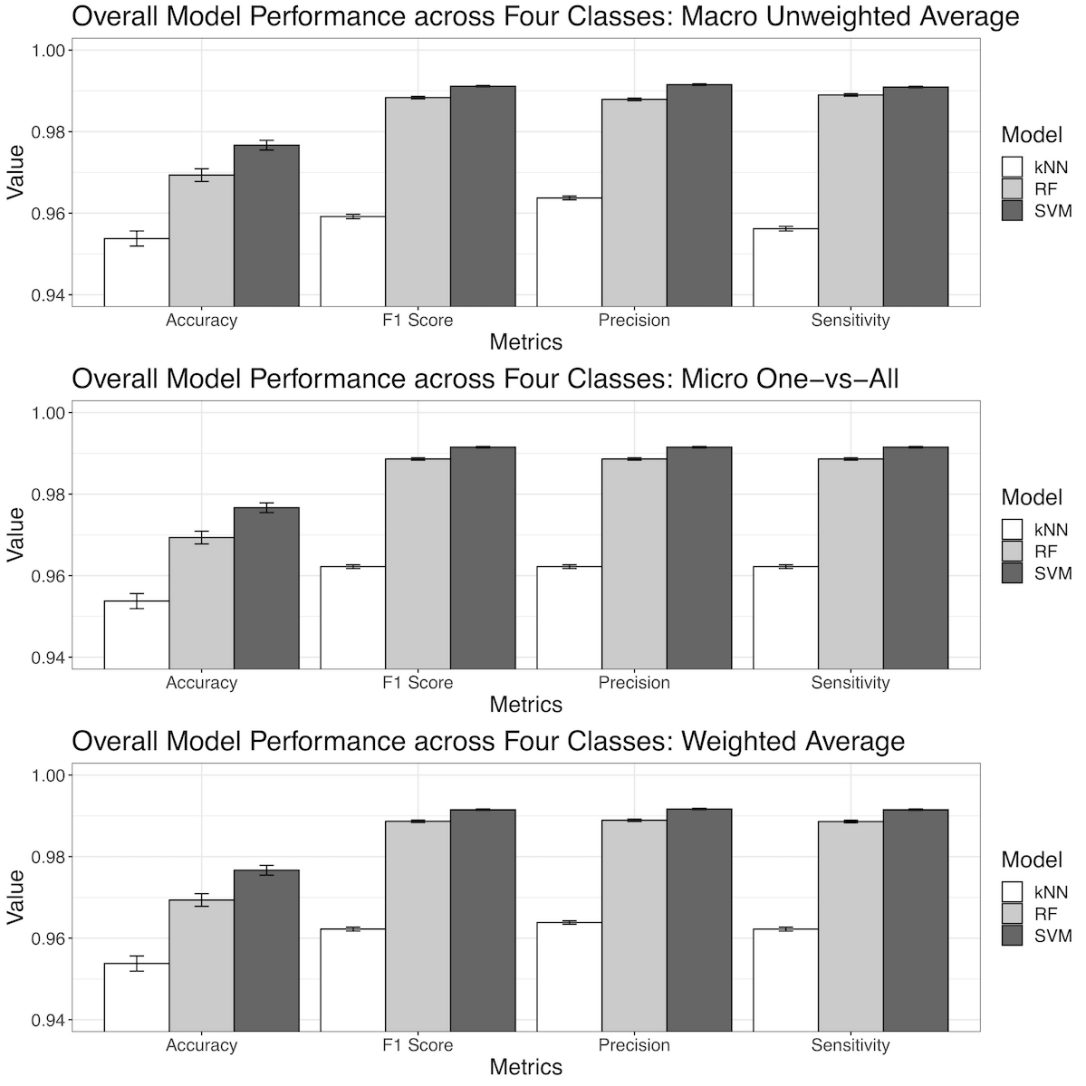
The overall performance of machine learning models across the 4 classes. Model performance was based on the prediction of unseen testing data (ie, the 20% of the original data), not on the 80% of the original data that were used to develop the model. kNN: k nearest neighbor; RF: random forest; SVM: support vector machine.

We developed and validated 3 different types of ML models, as follows: the kNN, RF, and SVM models. With regard to training data, the average overall multimodal classification accuracy of the kNN, RF, and SVM models was 96.2% (SE 0.5%), 99.8% (SE 0.3%), and 99.2% (SE 0.2%), respectively. With regard to test data, the average overall multimodal classification accuracy of the 3 models was 95.4% (SE 0.2%), 96.9% (SE 0.2%), and 97.7% (SE 0.1%), respectively (Figure 5). These 3 models also achieved consistent and high performance across all 4 classes based on the different approaches for calculating the overall performance, including the micro approach (ie, the one-vs-all approach), macro approach (ie, unweighted averages across all 4 classes), and weighted average approach (ie, based on percentage of each class in the entire sample). It should be noted that overall accuracy did not depend on sample size, so there was only 1 approach for calculating accuracy. The F1 score, sensitivity, and precision were quantified via each approach (ie, the micro, macro, and weighted average approaches). The F1 scores that were calculated using the macro approach were 95.9% (SE 0.1%), 98.8% (SE<0.1%), and 99.1% (SE<0.1%) for the kNN, RF, and SVM models, respectively. The F1 scores that were calculated using the micro approach was 96.2% (SE<0.1%), 98.8% (SE<0.1%), and 99.2% (SE<0.1%) for the kNN, RF, and SVM models, respectively. The F1 scores calculated using the weighted average approach was 96.2% (SE<0.1%), 98.9% (SE<0.1%), and 99.2% (SE<0.1%) for the kNN, RF, and SVM models, respectively. The differences in F1 scores based on the different approaches (ie, the micro, macro, and weighted average approaches) were minimal (Figure 5). In addition, the differences in F1 scores across the different ML models (Figure 5) were also not significant. Similarly, model sensitivity and precision were all >95% for all ML model types and all approaches for calculating the performance metric. The complete overall performance metrics for the 3 different evaluation approaches and 3 ML models are presented in Multimedia Appendix 7.

After examining the performance metrics across the 3 different types of ML models, it was clear that the SVM model consistently had the best performance with regard to all metrics, followed by the RF model, though the difference was almost indistinguishable. The kNN model had about a 1%-3% deficiency in performance compared to the other 2 models. It should be noted that the kNN model also had an accuracy, F1 score, sensitivity, and precision of at least 95%. Therefore, the kNN model was only bested by 2 even more competitive models. Furthermore, the relatively small standard errors demonstrated that the ML models were robust against different randomly sampled inputs (Multimedia Appendix 7).

**Figure 6:**
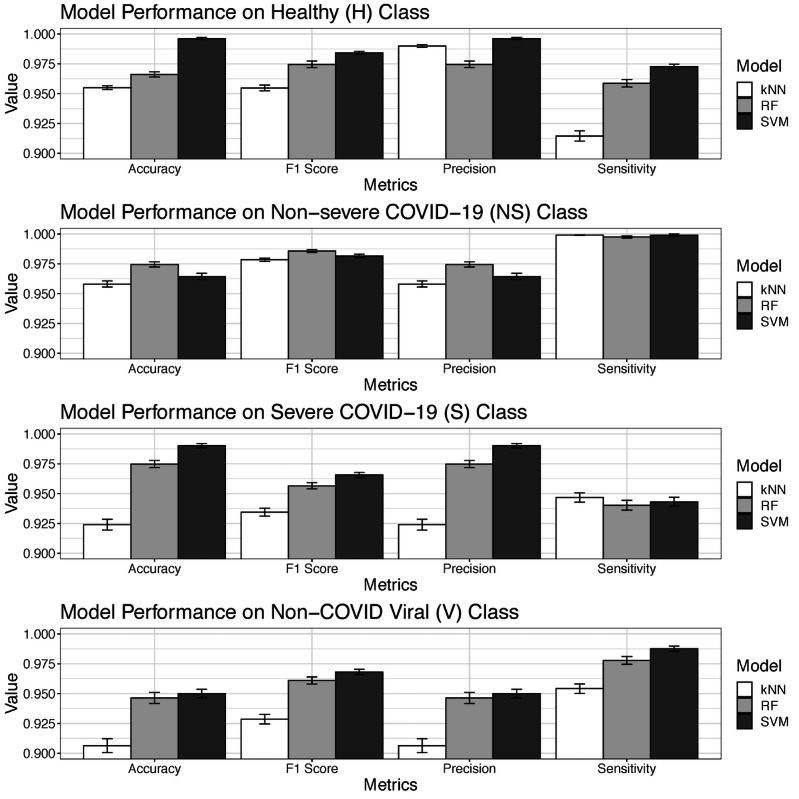
Class-specific performance of machine learning models. kNN: k nearest neighbor; RF: random forest; SVM: support vector machine.

With regard to each individual class, the noninfected healthy class had a 95.2%-99.9% prediction accuracy, 95.5%-98.4% F1 score, 91.4%-97.3% sensitivity, and 97.5%-99.9% precision in the testing set, depending on the specific ML model used. It should be noted these are ranges, not standard errors, as shown in Figure 6. The approach to computing class-specific model performance was the one-vs-all approach. With regard to the nonsevere COVID-19 class, ML models achieved a 95.8%-97.4% accuracy, 97.8%-98.6% F1 score, 99.8%-99.9% sensitivity, and 95.8%-97.4% precision. With regard to the severe COVID-19 class, ML models achieved a 92.4%-99.0% accuracy, 93.4%-96.6% F1 score, 94.3%-94.7% sensitivity, and 92.4%-99.0% precision. With regard to the non-COVID viral pneumonia infection class, ML models achieved a 90.6%-95.0% accuracy, 92.9%-96.8% F1 score, 95.4%-98.8% sensitivity, and 90.6%-95.0% precision. The non-COVID viral infection class was relatively more challenging to differentiate from the other 3 classes, but the difference was not substantial. Therefore, the potential clinical use of the ML models is still justified. Similar to the results of overall model performance (Figure 5), class-specific performance metrics also had relatively small standard errors, indicating that the training of models was consistent and robust against randomly selected inputs. Except for a few classes and model performance metrics, the SVM model performed slightly better than the RF and kNN models. The complete class-specific results are shown in Figure 6. The complete class-specific performance metrics across the 3 ML models are shown in Multimedia Appendix 8.

All 3 ML multinomial classification models, which were based on different computational techniques, had consistently high overall performance (Figure 5, Table S3) and high performance for each specific class (Figure 6, Multimedia Appendix 8). Of the 3 types of ML models developed and evaluated, the SVM model was marginally better than the RF and kNN models. As a result, the ML multinomial classification models were able to accurately differentiate between the 4 classes all at once, provide accurate and detailed class-specific predictions, and act as reliable decision-making tools for clinical diagnostic support and the triaging of patients with suspected COVID-19, who might or might not be infected with a clinically similar type of virus other than SARS-CoV-2.

In addition to the multimodal classification that incorporated all 3 different feature sets (ie, binary clinical, continuous lab testing, and CT features in the ResNet CNN; Figure 1), we also tested how each specific feature modality performed without feature fusion (ie, unimodality). By using each of the 23 symptom features alone, the RF, kNN, and SVM models achieved an average accuracy of 74.5% (SE 0.3%), 73.3% (SE 0.3%), and 75.5% (SE 0.3%) with the testing set, respectively. By using each of the 10 lab testing features alone, the RF, kNN, and SVM models achieved an average accuracy of 67.7% (SE 0.4%), 56.2% (SE 0.4%), and 59.5% (SE 0.3%) with the testing set, respectively.

The overall accuracy of the CNN with CT scan data alone was 90.8% (SE 0.3%) across the 4 classes. With regard to each pair of classes, the CNN was able to accurately differentiate between the severe COVID-19 and noninfected healthy classes with 99.9% (SE<0.1%) accuracy, the non-COVID viral infection and noninfected healthy classes with 99.2% (SE 0.1%) accuracy, the severe COVID-19 and nonsevere COVID-19 classes with 95.4% (SE 0.1%) accuracy, and the non-severe COVID-19 and noninfected healthy classes with 90.3% (SE 0.2%) accuracy. However, by using CT features alone (ie, without feature late fusion), the CNN could only differentiate between the non-COVID viral infection and nonsevere COVID-19 classes with 84.9% (SE 0.2%) accuracy, and the non-COVID viral infection and severe COVID-19 with 74.2% (SE 0.2%) accuracy in the testing set.

Substantial performance boosts were gained by combining input features from the different feature modalities and performing multimodal classification, instead of using a single-feature modality alone. A 15%-42% increase in prediction accuracy with the testing set was achieved compared to the single-modality models. It should be noted that the RF, SVM, and kNN models were technically distinct ML models. However, the performance differences between these 3 distinct ML models were marginal, based on the multimodal features. Therefore, we concluded that the high performance in COVID-19 classification in this study (Figures 5 and 6) was largely due to multimodal feature late fusion, not due to the specific type of ML model.

Gini impurity scores derived from the RF model identified major contributing factors that differentiated the 4 classes. With regard to clinical feature modality, the top 3 most influential features were fever, coughing, and old age (ie, >50 years). The forest plots of odds ratios for these features are provided in Figure 2, which shows the exact influence that these features had across classes. With regard to lab testing features, the top 3 most influential features, in descending order, were high-sensitivity C-reactive protein level, hemoglobin level, and absolute neutrophil count. The distribution of these 3 features across the 4 classes and the results of multiple comparisons are shown in Figure 3. Although high-sensitivity C-reactive protein level is a known factor for COVID-19 severity and prognosis [42], we showed that it could also differentiate patients with COVID-19 from patients with non-COVID viral pneumonia and healthy individuals. In addition, we learned that different hemoglobin and neutrophil levels were novel features for accurately distinguishing between patients with clinical COVID-19, patients with non-COVID viral pneumonia, and healthy individuals. These results shed light on which set of clinical and lab testing features are the most critical in identifying COVID-19, which will help guide clinical practice. With regard to the CT features extracted from the CNN, the RF models identified the top 3 influential features, which were CT features 6, 10, and 1 in the 10-element FC2 layer (Figure 4). Although the actual clinical interpretation of CT features was not clear at the time of this study due to the nature of DL models, including the ResNet CNN applied in this study, these features showed promise in accurately differentiating between multinomial classes all at once via CT scans, instead of training several CNNs for binary classifications between each class pair. Future research might reveal the clinical relevance of these features in a more interpretable way with COVID-19 pathology data.

## Discussion

### Principal Findings

In this study, we provided a more holistic perspective to characterizing COVID-19 and accurately differentiating COVID-19, especially nonsevere COVID-19, from other clinically similar viral pneumonias and noninfections. The human body is an integrated and systemic entity. When the body is infected by pathogens, clinical consequences can be detected not only with biomedical imaging features (eg, CT scan features), but also with other features, such as lab testing results for blood biochemistry [20,43]. A single-feature modality might not reveal the full clinical consequences and provide the best predictive power for COVID-19 detection and classification, but the synergy of multiple modalities exceeds the power of any single modality. Currently, multimodality medical data can be effectively stored, transferred, and exchanged with electronic health record systems. The economic cost of acquiring clinical and lab testing modality data are lower than the economic cost of acquiring current confirmatory qRT-PCR data. Availability and readiness are also advantages that these modalities have over qRT-PCR, which currently has a long turnaround time. This study harnessed the power of multimodality medical information for an emerging pandemic, for which confirmatory molecular tests have reliability and availability issues across time and space. This study’s novel analytical framework can be used to prepare for incoming waves of disease epidemics in the future, when clinicians’ experience and understanding with the disease may vary substantially.

Upon the further examination of comprehensive patient symptom data, we believed that our current understanding and definition of asymptomatic COVID-19 would be inadequate. Of the 214 patients with nonsevere COVID-19, 60 (28%) had no fever (ie, <37°C), 78 (36.4%) did not experience coughing, 141 (65.9%) did not feel chest congestion and pain, and 172 (80.4%) did not report having a sore throat upon admission. Additionally, there were 10 (4.7%) patients with confirmed COVID-19 in the nonsevere COVID-19 class who did not present with any of these common symptoms and could be considered patients with asymptomatic COVID-19. Even after considering headache, muscle pain, and fatigue, there were still 4 (1.9%) patients who did not show symptoms related to typical respiratory diseases. Of these 4 patients, 1 (25%) had diarrhea upon admission. Therefore, using symptom features alone is not sufficient for detecting and differentiating patients with asymptomatic COVID-19. Nevertheless, all asymptomatic patients were successfully detected via our model, and no false negatives were observed. This finding shows the incompleteness of the current definition and understanding of asymptomatic COVID-19, and the potential power that nontraditional analytical tools have for identifying these patients.

Based on this perspective, we developed a comprehensive end-to-end analytical framework that integrated both high-dimensional biomedical imaging data and low-dimensional clinical and lab testing data. CT scans were first processed with DL CNNs. We developed a customized ResNet CNN architecture with 2 FC layers before the final output layer. We then used the second FC layer as the low-dimensional representation of the original high-dimensional CT data. In other words, a CNN was applied first for dimensional reduction. The feature fusion of CT (ie, represented by the FC layers), clinical, and lab testing feature modalities demonstrated feasibility and high accuracy in differentiating between the nonsevere COVID-19, severe COVID-19, non-COVID viral pneumonia, and noninfected healthy classes all at once. The consistent high performance across the 3 different types of ML models (ie, the RF, SVM, and kNN models), as well as the substantial performance boost from using a single modality, further unleashed the hidden power of feature fusion for different biomedical feature modalities. Compared to the accuracy of using any single-feature modality alone (60%-80%), the feature fusion of multimodal biomedical data substantially boosted prediction accuracy (>97%) in the testing set.

We compared the performance of our model, which was based on the multimodal biomedical data of 683 participants, against the performance of state-of-the-art benchmarks in COVID-19 classification studies. A DL study that involved thoracic CT scans for 87 participants claimed to have >99% accuracy [37], and another study with 200 participants claimed to have 86%-99% accuracy in differentiating between individuals with and without COVID-19 [36]. Another study reported a 95% area under the curve for differentiating between COVID-19 and other community-acquired pneumonia diseases in 3322 participants [39]. Furthermore, a 92% area under the curve was achieved in a study of 905 participants with and without COVID-19 by using multimodal CT, clinical, and lab testing information [44]. A study that used CT scans to differentiate between 3 multinomial classes (ie, the COVID [no clinical state information], non-COVID viral pneumonia, and healthy classes) achieved an 89%-96% accuracy based on a total of 230 participants [38]. In addition, professionally trained human radiologists have achieved a 60%-83% accuracy in differentiating COVID-19 from other types of community-acquired pneumonia diseases [45]. Therefore, the performance of our model is on par with, or superior to, the performance of these benchmark models and exceeds the performance of human radiologists. Moreover, previous studies have generally focused on differentiating patients with COVID-19 from individuals without COVID-19 or patients with other types of pneumonia. In other words, the current COVID-19 classification models are mostly binary classifiers. Our study not only detected COVID-19 in healthy individuals, but also addressed the more important clinical issue of differentiating COVID-19 from other viral infections. Our study also distinguished between different COVID-19 clinical states (ie, severe vs nonsevere). Therefore, our study provides a novel and effective breakthrough for clinical applications, not just incremental improvements for existing ML models.

The success of this study sheds light on many other disease systems that use multimodal biomedical data inputs. Specifically, the feature fusion of high- and low-dimensional biomedical data modalities can be applied to more feature modalities, such as individual-level high-dimensional “-omics” data. Currently, a study on the genome-wide association between individual single nucleotide polymorphisms and COVID-19 susceptibility has revealed several target loci that are involved in COVID-19 pathology. Following a similar approach, we may also conduct another study, in which we first carry out the dimensional reduction of “-omics” data, and then perform data fusion with other low-dimensional modalities [46-48].

With regard to classification, this study adopts a hybrid of DL (ie, CNN) and ML (ie, RF, SVM, and kNN ML) models via feature late fusion. By using various data-driven methods, we avoided the potential cause-effect pitfall and focused directly on the more important clinical question. For instance, many comorbidities, such as diabetes [49,50] and cardiovascular diseases [51,52], are strongly associated with the occurrence of severe COVID-19. It is still unclear whether diabetes or reduced kidney function causes severe COVID-19, whether SARS-CoV-2 infection worsens existing diabetes, or whether diabetes and COVID-19 actually mutually influence each other and result in undesirable clinical prognoses. Future studies can use data-driven methods to further investigate the causality of comorbidities and COVID-19.

There are some limitations in this study and potential improvements for future research. For instance, to perform multinomial classification across the 4 classes, we had to discard a lot of features, especially those in the lab testing modality. The non-COVID viral pneumonia class used a different electronic health record system that collected different lab testing features from participants in Wuhan (ie, participants in the severe COVID-19, nonsevere COVID-19, and noninfected healthy classes). Many lab testing features were able to accurately differentiate between severe and nonsevere COVID-19 in our preliminary study, such as high-sensitivity Troponin I level, D-dimer level, and lactate dehydrogenase level. However, these features were not present, or largely missing, in the non-COVID viral infection class. Eventually, only 10 lab testing features were included, which is small compared to the average of 20-30 features that are usually available in different electronic health record systems. This is probably the reason why the lab testing feature modality alone was not able to provide accurate classifications (ie, the highest accuracy achieved was 67.7% with the RF model) across all 4 classes in this study. In addition, although we had a reasonably large participant pool of 638 individuals, more participants are needed to further validate the findings of this study.

Another potential practical pitfall was that not all feature modalities were readily available at the same time for feature fusion and multimodal classification. With regard to single-modality features, CT had the best performance in generating accurate predictions. However, CT is usually performed in the radiology department. Lab testing may be outsourced, and obtaining lab test results takes time. Consequently, there might be lags in data availability among different feature modalities. We believe that when multimodal features are not available all at once, single-modality features can be used to perform first-round triaging. Multimodal features are needed when accuracy is a must.

It should be noted that although the participants in this study came from different health care facilities, the majority of them were of Chinese Han ethnicity. The biomedical features among the different COVID-19 and non-COVID classes may be different in people of other races and ethnicities, or people with other confounding factors. The cross-validation of the findings in this study based on other ethnicity groups and larger sample sizes is needed for future research.

This study used a common CNN architecture (ie, a ResNet). The 10 CT features extracted from the FC2 layer of the ResNet were used to match the dimensionality of the other 2 low-dimensional feature modalities. Future research on different disease systems can explore and compare other architectures that use different biomedical imaging data (eg, CT, X-ray, and histology data). The actual dimensionality of the FC2 layer can also be optimized to deliver better performance. Finally, this study presented the results of individual classification models. To achieve even higher performance, the combination of multiple models can be explored in future studies.

### Conclusion

In summary, different biomedical information across different modalities, such as clinical information, lab testing results, and CT scans, work synergistically to reveal the multifaceted nature of COVID-19 pathology. Our ML and DL models provided a feasible technical method for working directly with multimodal biomedical data and differentiating between patients with severe COVID-19, patients with nonsevere COVID-19, patients with non-COVID viral infection, and noninfected healthy individuals at the same time, with >97% accuracy.

## Appendix

Multimedia Appendix 1 Demographic, clinical, and lab testing results of the 4 Classes.

Multimedia Appendix 2 Architecture of the customized ResNet-18 CNN and sample computed tomography scans of the 4 classes. CNN: convolutional neural networkl ResNet: residual neural network.

Multimedia Appendix 3 Comparison of clinical features across the 4 classes.

Multimedia Appendix 4 Comparison of lab testing features across the 4 classes.

Multimedia Appendix 5 Computed tomography features extracted via a deep learning convolutional neural network and compared across the 4 classes.

Multimedia Appendix 6 z-test and Kolmogorov-Smirnov test results of significance for each biomedical feature among the 4 classes.

Multimedia Appendix 7 Overall machine learning model performance comparison.

Multimedia Appendix 8 Class-specific machine learning model performance comparison.

## Abbreviations

CT: computed tomography
CNN: convolutional neural network
DL: deep learning
DICOM: Digital Imaging and Communications in Medicine
FC: fully connected
GGO: ground-glass opacity
kNN: k-nearest neighbor
MERS: Middle East respiratory syndrome
ML: machine learning
qRT-PCR: quantitative real-time polymerase chain reaction
RF: random forest
ResNet: residual neural network
SARS: severe acute respiratory syndrome
SVM: support vector machine
