# Supplementary material for: Accurately Differentiating Between Patients With COVID-19, Patients With Other Viral Infections, and Healthy Individuals: Multimodal Late Fusion Learning Approach
Source: J Med Internet Res. 2021 Jan 6;23(1):e25535. doi: 10.2196/25535 (PMC7790733; doi:10.2196/25535)
Supplement: Multimedia Appendix 1 [file jmir_v23i1e25535_app1.docx]

**Table S1. Demographic, Clinical, and Lab Testing Results among Four Classes**

| Features/  Class | Non-Severe COVID | Severe-COVID | Viral Pneumonia | Healthy |
| --- | --- | --- | --- | --- |
| Demographic | |  |  |  |
| Participant | 214 | 142 | 129 | 198 |
| Male | 132 | 62 | 72 | 101 |
| Female | 82 | 80 | 57 | 97 |
| Age | 48±15 | 68±13 | 52±17 | 38±10 |
| Clinical (%) |  |  |  |  |
| SMK | 3.27 | 5.41 | 16.28 | 0 |
| HYP | 20.09 | 57.43 | 24.03 | 0 |
| DIA | 8.41 | 29.73 | 5.43 | 0 |
| CAR | 5.61 | 25.00 | 10.85 | 0 |
| CPD | 0.00 | 4.73 | 0.78 | 0 |
| FEV | 70.09 | 74.32 | 47.29 | 0 |
| LOF | 33.18 | 30.41 | 13.18 | 0 |
| MDF | 23.36 | 26.35 | 14.73 | 0 |
| HIF | 11.21 | 16.89 | 19.38 | 0 |
| SOR | 19.63 | 6.76 | 6.98 | 0 |
| COU | 63.55 | 63.51 | 89.92 | 0 |
| MUC | 24.30 | 19.59 | 75.97 | 0 |
| HED | 11.21 | 6.08 | 4.65 | 0 |
| CHL | 8.41 | 16.89 | 10.08 | 0 |
| MSA | 19.16 | 15.54 | 0.78 | 0 |
| FTG | 39.25 | 43.92 | 7.75 | 0 |
| SHB | 34.11 | 49.32 | 9.30 | 0 |
| DIR | 12.15 | 12.16 | 0.00 | 0 |
| NAP | 7.94 | 21.62 | 1.55 | 0 |
| VOM | 5.61 | 2.03 | 10.85 | 0 |
| Lab Testing (Mean±SE) |  |  |  |  |
| WBC | 5.05±0.12 | 6.81±0.15 | 5.77±0.14 | 5.73±0.09 |
| HGB | 125.22±1.18 | 122.59±1.42 | 124.98±1.16 | 142.79±1.27 |
| PLT | 217.98±5.85 | 216.91±9.03 | 223.07±7.45 | 228.58±4.01 |
| NE% | 64.22±3.36 | 59.78±4.04 | 63.24±0.82 | 56.28±0.52 |
| NE | 3.13±0.10 | 4.50±0.12 | 3.68±0.11 | 3.25±0.07 |
| LY% | 28.51±0.74 | 21.41±0.89 | 26.49±0.73 | 34.25±0.56 |
| LY | 1.36±0.04 | 1.45±0.05 | 1.49±0.05 | 1.94±0.04 |
| CRP | 20.68±2.00 | 41.93±2.41 | 1.74±0.18 | 0.91±0.09 |
| TBIL | 11.66±0.37 | 14.36±0.44 | 10.27±0.40 | 14.17±0.37 |
| CREA | 93.32±24.03 | 86.82±28.89 | 63.28±3.89 | 69.57±1.16 |
