# Supplementary figures and images for: Accurately Differentiating Between Patients With COVID-19, Patients With Other Viral Infections, and Healthy Individuals: Multimodal Late Fusion Learning Approach
Source: J Med Internet Res. 2021 Jan 6;23(1):e25535. doi: 10.2196/25535 (PMC7790733; doi:10.2196/25535)

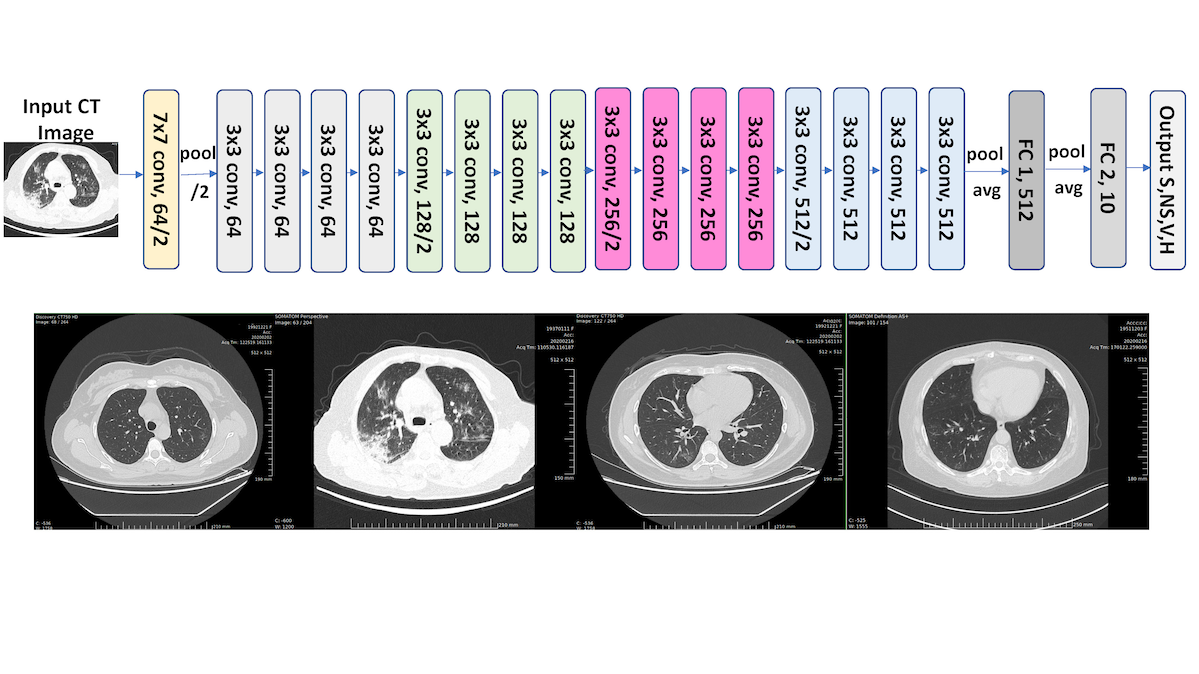

Supplement: Multimedia Appendix 2 [file jmir_v23i1e25535_app2.png]

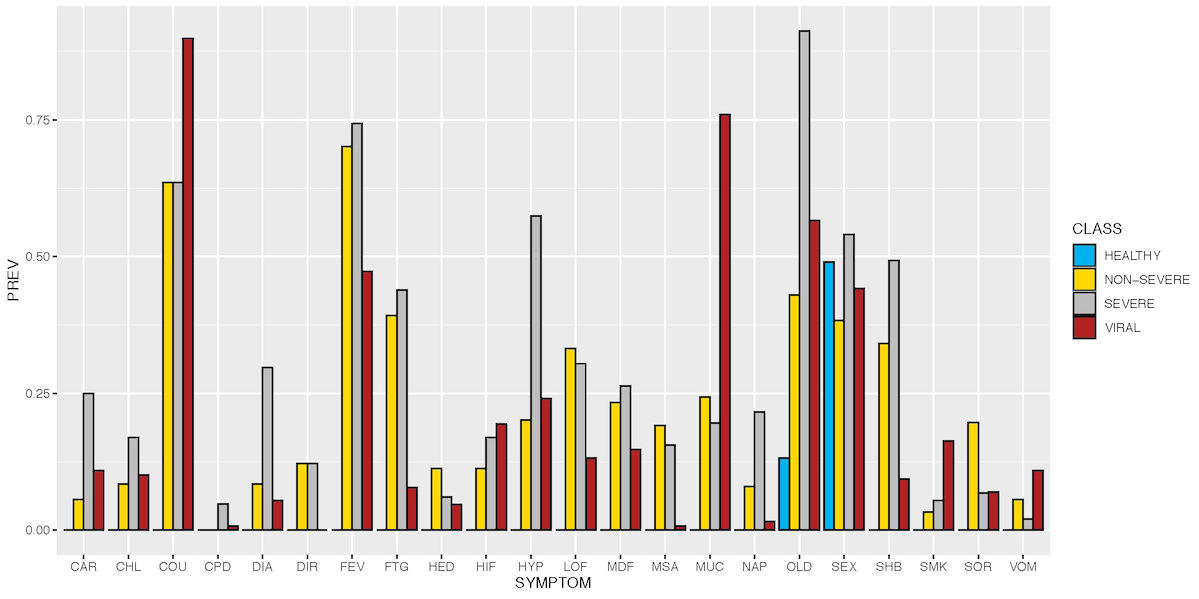

Supplement: Multimedia Appendix 3 [file jmir_v23i1e25535_app3.png]

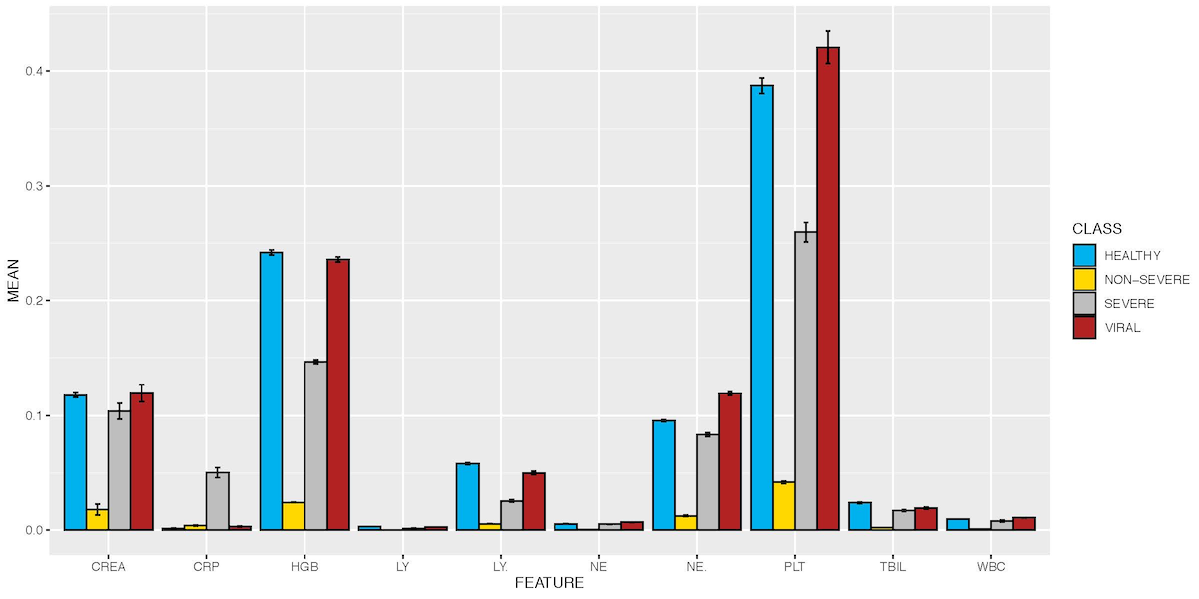

Supplement: Multimedia Appendix 4 [file jmir_v23i1e25535_app4.png]

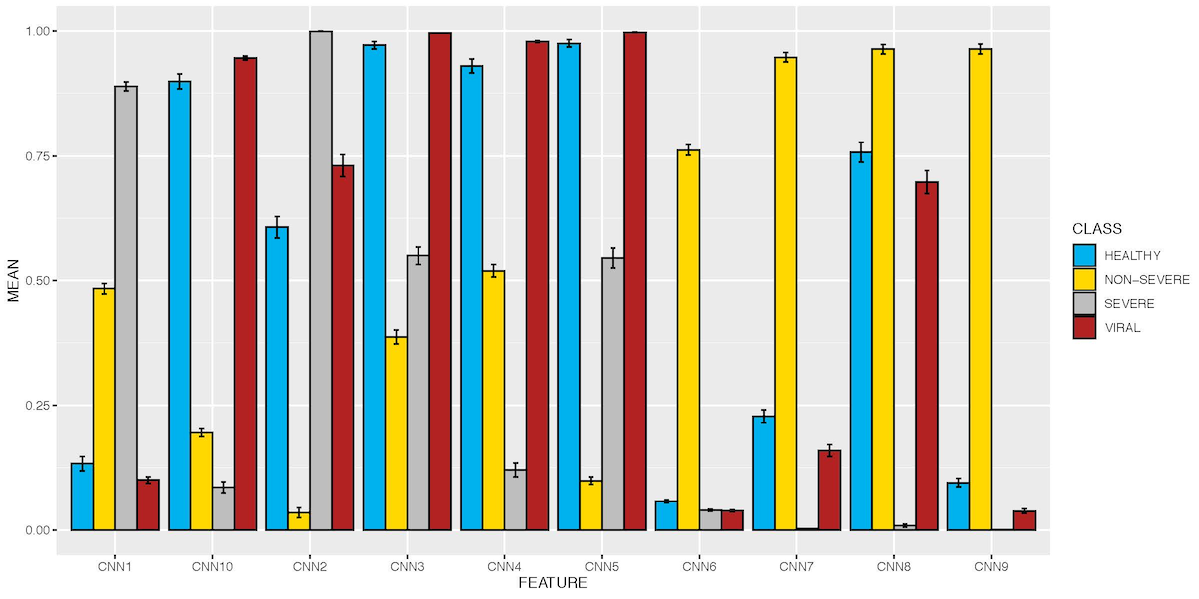

Supplement: Multimedia Appendix 5 [file jmir_v23i1e25535_app5.png]
