# Supplementary material for: Accurately Differentiating Between Patients With COVID-19, Patients With Other Viral Infections, and Healthy Individuals: Multimodal Late Fusion Learning Approach
Source: J Med Internet Res. 2021 Jan 6;23(1):e25535. doi: 10.2196/25535 (PMC7790733; doi:10.2196/25535)
Supplement: Multimedia Appendix 6 [file jmir_v23i1e25535_app6.docx]

**Table S2. Z-Test and/or Kolmogorov-Smirnov Test Results of Significance for Each Biomedical Feature among Four Classes**

| Features/  Class Pair | NS-S | NS-H | NS-V | S-H | S-V | V-H |
| --- | --- | --- | --- | --- | --- | --- |
| Clinical  (Z-Test) | |  |  |  |  |  |
| SMK | 0.463 | 0.029 | <0.001 | 0.003 | 0.006 | <0.001 |
| HYP | <0.001 | <0.001 | 0.470 | <0.001 | <0.001 | <0.001 |
| DIA | <0.001 | <0.001 | 0.415 | <0.001 | <0.001 | 0.003 |
| CAR | <0.001 | 0.002 | 0.117 | <0.001 | 0.004 | <0.001 |
| CPD | 0.005 | NA | 0.798 | 0.007 | 0.109 | 0.829 |
| FEV | 0.447 | <0.001 | <0.001 | <0.001 | <0.001 | <0.001 |
| LOF | 0.659 | <0.001 | <0.001 | <0.001 | 0.001 | <0.001 |
| MDF | 0.600 | <0.001 | 0.073 | <0.001 | 0.026 | <0.001 |
| HIF | 0.163 | <0.001 | 0.053 | <0.001 | 0.704 | <0.001 |
| SOR | 0.001 | <0.001 | 0.002 | 0.001 | 1.000 | 0.001 |
| COU | 1.000 | <0.001 | <0.001 | <0.001 | <0.001 | <0.001 |
| MUC | 0.354 | <0.001 | <0.001 | <0.001 | <0.001 | <0.001 |
| HED | 0.138 | <0.001 | 0.059 | 0.002 | 0.796 | 0.008 |
| CHL | 0.022 | <0.001 | 0.744 | <0.001 | 0.142 | <0.001 |
| MSA | 0.455 | <0.001 | <0.001 | <0.001 | <0.001 | 0.829 |
| FTG | 0.436 | <0.001 | <0.001 | <0.001 | <0.001 | <0.001 |
| SHB | 0.005 | <0.001 | <0.001 | <0.001 | <0.001 | <0.001 |
| DIR | 1.000 | <0.001 | <0.001 | <0.001 | <0.001 | NA |
| NAP | <0.001 | <0.001 | 0.024 | <0.001 | <0.001 | 0.302 |
| VOM | 0.158 | 0.002 | 0.117 | 0.154 | 0.005 | <0.001 |
| OLD | <0.001 | <0.001 | 0.020 | <0.001 | <0.001 | <0.001 |
| SEX | 0.004 | 0.037 | 0.338 | 0.410 | 0.129 | 0.461 |
| Lab Testing  (KS-Test) | |  |  |  |  |  |
| WBC | 0.024 | <0.001 | <0.001 | 0.001 | 0.014 | 0.696 |
| HGB | 0.744 | <0.001 | 0.653 | <0.001 | 0.113 | <0.001 |
| PLT | 0.614 | <0.001 | 0.143 | <0.001 | 0.020 | 0.020 |
| NE. | <0.001 | <0.001 | <0.001 | <0.001 | <0.001 | <0.001 |
| NE | <0.001 | 0.004 | <0.001 | <0.001 | 0.082 | 0.013 |
| LY. | <0.001 | <0.001 | 0.038 | <0.001 | <0.001 | <0.001 |
| LY | 0.002 | <0.001 | 0.039 | <0.001 | <0.001 | <0.001 |
| CRP | <0.001 | <0.001 | <0.001 | <0.001 | <0.001 | <0.001 |
| TBIL | 0.001 | <0.001 | 0.088 | 0.236 | <0.001 | <0.001 |
| CREA | <0.001 | 0.091 | <0.001 | 0.013 | <0.001 | <0.001 |
| CT CNN  (KS-Test) | |  |  |  |  |  |
| CNN1 | <0.001 | <0.001 | <0.001 | <0.001 | <0.001 | 0.254 |
| CNN2 | <0.001 | <0.001 | <0.001 | <0.001 | <0.001 | 0.012 |
| CNN3 | <0.001 | <0.001 | <0.001 | <0.001 | <0.001 | 0.021 |
| CNN4 | <0.001 | <0.001 | <0.001 | <0.001 | <0.001 | 0.163 |
| CNN5 | <0.001 | <0.001 | <0.001 | <0.001 | <0.001 | 0.147 |
| CNN6 | <0.001 | <0.001 | <0.001 | <0.001 | 0.099 | <0.001 |
| CNN7 | <0.001 | <0.001 | <0.001 | <0.001 | <0.001 | 0.011 |
| CNN8 | <0.001 | <0.001 | <0.001 | <0.001 | <0.001 | 0.008 |
| CNN9 | <0.001 | <0.001 | <0.001 | <0.001 | <0.001 | <0.001 |
| CNN10 | <0.001 | <0.001 | <0.001 | <0.001 | <0.001 | 0.006 |

Note: P value based on Z-test for clinical features and Kolmogorov-Smirnov KS-test for lab testing and CT CNN features.

| *P*≤0.05; | *P*≤0.01; | *P*≤0.001 |
| --- | --- | --- |
