# Supplementary material for: Accurately Differentiating Between Patients With COVID-19, Patients With Other Viral Infections, and Healthy Individuals: Multimodal Late Fusion Learning Approach
Source: J Med Internet Res. 2021 Jan 6;23(1):e25535. doi: 10.2196/25535 (PMC7790733; doi:10.2196/25535)
Supplement: Multimedia Appendix 7 [file jmir_v23i1e25535_app7.docx]

**Table S3. Overall Machine Learning Model Performance Comparison**

| **Model/Method** | | **Macro Unweighted Average** | | **Micro One-vs-All** | | **Weighted Average** | |
| --- | --- | --- | --- | --- | --- | --- | --- |
|  | **Metric** | **Mean** | **SE** | **Mean** | **SE** | **Mean** | **SE** |
| **RF** | **Accuracy** | 0.969 | 0.002 | 0.969 | 0.002 | 0.969 | 0.002 |
| **kNN** | **Accuracy** | 0.954 | 0.002 | 0.954 | 0.002 | 0.954 | 0.002 |
| **SVM** | **Accuracy** | 0.977 | 0.001 | 0.977 | 0.001 | 0.977 | 0.001 |
| **RF** | **F1 Score** | 0.988 | 0.000 | 0.989 | 0.000 | 0.989 | 0.000 |
| **kNN** | **F1 Score** | 0.959 | 0.001 | 0.962 | 0.000 | 0.962 | 0.000 |
| **SVM** | **F1 Score** | 0.991 | 0.000 | 0.992 | 0.000 | 0.992 | 0.000 |
| **RF** | **Sensitivity** | 0.989 | 0.000 | 0.989 | 0.000 | 0.989 | 0.000 |
| **kNN** | **Sensitivity** | 0.956 | 0.001 | 0.962 | 0.000 | 0.962 | 0.000 |
| **SVM** | **Sensitivity** | 0.991 | 0.000 | 0.992 | 0.000 | 0.992 | 0.000 |
| **RF** | **Precision** | 0.988 | 0.000 | 0.989 | 0.000 | 0.989 | 0.000 |
| **kNN** | **Precision** | 0.964 | 0.000 | 0.962 | 0.000 | 0.964 | 0.000 |
| **SVM** | **Precision** | 0.992 | 0.000 | 0.992 | 0.000 | 0.992 | 0.000 |
