# Supplementary material for: Accurately Differentiating Between Patients With COVID-19, Patients With Other Viral Infections, and Healthy Individuals: Multimodal Late Fusion Learning Approach
Source: J Med Internet Res. 2021 Jan 6;23(1):e25535. doi: 10.2196/25535 (PMC7790733; doi:10.2196/25535)
Supplement: Multimedia Appendix 8 [file jmir_v23i1e25535_app8.docx]

**Table S4. Class-specific Machine Learning Model Performance Comparison**

| **Model/Class** | | **Severe COVID-19 (S)** | | **Non-severe COVID-19 (NS)** | | **Non-COVID Viral Pneumonia (V)** | | **Non-Infected Healthy (H)** | |
| --- | --- | --- | --- | --- | --- | --- | --- | --- | --- |
|  | **Metric** | **Mean** | **SE** | **Mean** | **SE** | **Mean** | **SE** | **Mean** | **SE** |
| **RF** | **Accuracy** | 0.975 | 0.003 | 0.974 | 0.002 | 0.946 | 0.005 | 0.966 | 0.002 |
| **kNN** | **Accuracy** | 0.924 | 0.005 | 0.958 | 0.003 | 0.906 | 0.006 | 0.955 | 0.002 |
| **SVM** | **Accuracy** | 0.990 | 0.002 | 0.964 | 0.003 | 0.950 | 0.004 | 0.996 | 0.001 |
| **RF** | **F1 Score** | 0.957 | 0.003 | 0.986 | 0.001 | 0.961 | 0.003 | 0.975 | 0.003 |
| **kNN** | **F1 Score** | 0.934 | 0.003 | 0.978 | 0.001 | 0.929 | 0.004 | 0.955 | 0.002 |
| **SVM** | **F1 Score** | 0.966 | 0.002 | 0.982 | 0.001 | 0.968 | 0.002 | 0.984 | 0.001 |
| **RF** | **Sensitivity** | 0.940 | 0.004 | 0.998 | 0.001 | 0.978 | 0.003 | 0.959 | 0.003 |
| **kNN** | **Sensitivity** | 0.947 | 0.004 | 0.999 | 0.000 | 0.954 | 0.004 | 0.915 | 0.004 |
| **SVM** | **Sensitivity** | 0.943 | 0.004 | 0.999 | 0.001 | 0.988 | 0.002 | 0.973 | 0.002 |
| **RF** | **Precision** | 0.975 | 0.003 | 0.974 | 0.002 | 0.946 | 0.005 | 0.975 | 0.003 |
| **kNN** | **Precision** | 0.924 | 0.005 | 0.958 | 0.003 | 0.906 | 0.006 | 0.990 | 0.001 |
| **SVM** | **Precision** | 0.990 | 0.002 | 0.964 | 0.003 | 0.950 | 0.004 | 0.996 | 0.001 |
